# Post-traumatic responses to workplace violence among nursing professionals: a collaborative and comparative study in South Korea and Hong Kong

**DOI:** 10.1186/s12912-023-01502-7

**Published:** 2023-10-04

**Authors:** Soyun Hong, Sujin Nam, Janet Yuen Ha Wong, Heejung Kim

**Affiliations:** 1 College of Nursing Brain Korea 21 FOUR Project, Yonsei University, Seoul, Republic of Korea; 2 Department of Nursing, Korean Bible University, Seoul, Republic of Korea; 3 School of Nursing, Li Ka Shing Faculty of Medicine, The University of Hong Kong, Hong Kong, China; 4 School of Nursing and Health Studies, Hong Kong Metropolitan University, Hong Kong, China; 5 College of Nursing and Mo-Im Kim Nursing Research Institute, Yonsei University, Seoul, Republic of Korea

**Keywords:** Adaptation, Psychological, Mental health, Nurses, Post-traumatic growth, Psychological, Stress disorders, Post-traumatic, Workplace violence

## Abstract

**Background:**

Workplace violence has had a significant and negative psychological impact on nursing professionals worldwide. Concerted worldwide efforts to improve work environments have not yet removed nursing professionals from the threat of violence. It is highly essential to conduct comparative research in various working environments where the nurses of each country have unique experiences of workplace violence. The aim of this study was to examine the differences in the rate, associated factors, and post-traumatic responses to workplace violence between South Korean and Chinese nurses in Hong Kong among East Asian countries.

**Methods:**

A cross-sectional, correlational study design recruited a total of 471 registered nurses (319 South Korean nurses and 152 Chinese nurses in Hong Kong; overall response rate = 78.5%) at online communities in South Korea and Hong Kong. The data were collected by conducting a Qualtrics survey from January 15, 2020, to July 24, 2021. A structured questionnaire was administered for data collection, including rate of workplace violence, perception of workplace violence, attitudes toward workplace violence, coping styles, post-traumatic cognitions, post-traumatic stress disorder, post-traumatic growth, and mental health indicators (depression, anxiety, and stress). T-test, chi-squared, and binary logistic regression analyses were conducted.

**Results:**

In our sample, 30.7% South Korean nurses and 31.6% Chinese nurses in Hong Kong had experienced workplace violence. South Korean and Chinese nurses in Hong Kong with experience of workplace violence had lower perceptions of it. Nurses with experience of workplace violence reported lower levels of mental health, and this trend was more prominent among South Korean nurses.

**Conclusions:**

Our study findings showed a positive association between workplace violence and post-traumatic responses in both settings. We found that the close monitoring of post-traumatic responses associated with workplace violence could be improved by enhancing nurses’ perception of workplace violence.

## Background

Workplace violence against nursing professionals has been an enduring concern around the world [1]. Workplace violence refers to actions involving violence, encompassing everything from verbal abuse to physical attacks during duties [2]. Unequal power relationships contribute to a repetitive and persistent pattern of violent behavior in the workplace [1, 3, 4]. More specific forms of violence, such as nonphysical violence that are difficult to detect, are prevalent, making it difficult to identify work-related violence [5]. The World Health Organization emphasizes multidimensional aspects, including physical, sexual, verbal, and psychological abuse and workplace harassment [6]. Among the types of nonphysical violence, verbal abuse (58%) was the most common, followed by threats (33%) and sexual harassment (12%) [7]. The main perpetrators of health care workers were patients and visitors who lacked communication, especially in psychiatric and emergency department settings [7, 8]. Frontline nursing professionals experience excessive levels of stress because of poor working conditions, long shifts, and insufficient workforce during various medical emergency situations [9–11]. Registered nurses who reported being exposed to workplace violence experienced lower job performance and higher turnover intention, both of which are related to mental health problems [12, 13]. Thus, it is important to ensure the psychological and professional well-being of nurses who have experienced workplace violence.

Researchers have suggested that it is highly essential to conduct collaborative research in different countries, as the registered nurses of each country have unique experiences of workplace violence [14–16]. Moreover, previous studies have indicated that rates of exposure to workplace violence, the perpetrators, and the nature of violence vary across countries and cultures [14–16]. In a meta-analysis of 136 studies on workplace violence conducted with 151,347 nurses from a variety of settings, it was found that East Asian and Southeast Asia nurses face a higher level of physical violence from patient families/friends than those in Anglo and European regions [15]. Specifically, 47.5% of South Korean registered nurses had experienced workplace violence during a 1-month period, and the most common type of workplace violence was verbal abuse such as cursing, talking down, scramming, and threatening [17–21]. Of the Chinese registered nurses in Hong Kong, 44.6% have faced workplace violence during a 1-year period. Victimized nurses experienced a high level of verbal abuse and workplace harassment (39.2%) [22].

Based on Lazarus and Folkman’s [23] transactional stress and coping model, it can be understood that upon experiencing workplace violence, each nurse responds and copes differently. In the existing literature, workplace violence has been generally investigated in relation to mental health problems. For instance, after an incident of workplace violence, registered nurses experienced at least one symptom of post-traumatic stress, such as flashbacks, nightmares, avoidance, severe emotional distress, and physical reactions [12, 13, 24, 25]. However, there is a positive aspect to traumatic experiences (e.g., post-traumatic growth). Post-traumatic growth is defined as the positive psychological changes that may manifest in an individual after they have faced a challenging or adverse event [26]. Therefore, it is important to understand post-traumatic responses in both aspects, positive and negative, for mitigating workplace violence against nursing professionals.

Beyond the consequences of trauma, it is important to consider processes such as cognitive appraisal and coping to alleviate the impact of workplace violence on victims. A post-traumatic cognitive appraisal helps interpret the meanings of post-traumatic stress symptoms and growth [23]. Furthermore, a coping appraisal is closely associated with the choice of a coping style. Coping is defined as adapting an individual’s cognitive and behavioral efforts to their available resources during stressful situations [23]. Some studies have found different results related to the use of coping strategies for groups that have (or have not) experienced workplace violence [27, 28]. The group that has experienced workplace violence tends to use passive coping such as avoidance and denial, whereas the group that has not experienced it uses active coping styles such as confronting the perpetrator colleague or consulting the manager [27, 28]. Notably, passive coping styles increased mental health problems as compared to active coping [12, 27]. Therefore, it is necessary to examine nurses’ post-traumatic cognitive appraisal and coping styles when developing workplace violence interventions and organizational policies.

Chinese nurses in Hong Kong and South Korean nurses share several common environmental conditions and system characteristics. In Hong Kong, the health care system largely comprises public and private health care services, including hospitals and clinics [29]. The number of registered nurses in Hong Kong was 50,650 in 2022, including mainly Registered Nurses (General) (RNs(G)) (*n* = 46,935) and Registered Nurses (Psychiatric) (RNs(Psy)) (*n* = 3,704) [30]. In South Korea, the number of licensed registered nurses was 457,849 in 2021, with 215,817 of them working in hospitals, primarily general hospitals (*n* = 146,703) and hospitals (*n* = 65,516) [31]. Despite the differences in health care systems and environmental conditions between the two regions, the nurse-to-population ratio appears to be similar. Hong Kong had 8.7 registered nurses, including registered and enrolled nurses, per 1,000 populations in 2021 [32]. Korea had 8.8 practicing nurses per 1,000 populations in 2021 [33]. South Korea and Hong Kong are known to have similar age, sex ratio, and education levels for registered nurses [13, 22]. Several international studies have emphasized not only the importance of acknowledging the existence of workplace violence at the individual level but also the provision of appropriate systemic support at the cultural level. Hence, more collaborative research in similar cultures may enhance our understanding of workplace violence to create and propose international policies and guidelines.

### Conceptual framework

The conceptual framework of this study is based on the transactional stress and coping model of Lazarus and Folkman [23]. According to this framework, the adaptive responses of individuals who have experienced the same stressful stimulus will vary depending on their cognitive appraisal and coping styles: active coping (i.e., taking direct actions to address stressful situations) and passive coping (i.e., reducing the emotional distress arising from stressful situations). The model is based on the idea that stress responses may vary across individuals experiencing traumatic events such as a crisis, loss, and adversity depending on individuals’ efforts (i.e., problem- and emotion-focused coping styles). We hypothesized that, with exposure to workplace violence through a traumatic event, mental health would vary in registered nurses according to how they appreciated and coped in the specific culture based on a literature review [14–16] and the stress and coping theory.

### Objectives

In this study, we conducted a collaborative study on how Korean and Hong Kong nurses experience workplace violence by comparing how their responses to post-traumatic stress differ. The objectives of the study were (i) to examine the differences in the rate of workplace violence in two regions, (ii) to identify factors associated with exposure to workplace violence in two regions, and (iii) to compare post-traumatic responses to a violent experience in two regions.

## Methods

### Study design

This study was cross-sectional, correlational, and observational in design. We performed all methods in accordance with the relevant guidelines [34].

### Participants

A total of 578 nurses were recruited from online communities in South Korea and Hong Kong: 319 South Korean nurses and 152 Chinese nurses in Hong Kong. Of these, 471 nurses provided consent to participate in the study and completed the survey, and 107 nurses who did not meet the inclusion criteria or provide complete responses were excluded. The inclusion criteria were registered nurses who are currently working in a hospital and who provide direct nursing to patients in clinical settings, including general wards, specialized wards, outpatient clinics, and other units. The exclusion criterion was nurses with less than 6 months of nursing practice experience.

The required number of participants for statistical significance was 150 for each country. This number was estimated using G*Power 3.1, with effect size = .15, ⍺ = .05, power = .80, and 18 independent variables in a multivariate linear regression. Because the nonresponse rate for online surveys was approximately 50% [35], the goal was to recruit a total of 600 nurses from 300 South Korean nurses and 300 Chinese nurses in Hong Kong. The final sample in the analysis (*N* = 471) was sufficient to conduct a binary logistic regression.

### Data collection

We recognize that reporting on workplace violence through a face-to-face interview would be difficult, owing to the participants’ confidentiality and privacy concerns during the COVID-19 pandemic. Therefore, we considered an online survey to be a more appropriate method for data collection. After obtaining approval from the ethics committee of the university’s institutional review board of South Korea (approval no. Y-2019–0146) and Hong Kong (approval no. UW 19–571), data were collected between January 15, 2020, and July 24, 2021. Participants were recruited through online advertisements on the community’s communication bulletin board. Moreover, a few participants volunteered to complete the online survey via a Qualtrics survey on a computer or mobile device. The first page of the survey included an informed consent form that contained information on the study. Before presenting the main survey questions, participants were screened using three eligibility questions. The collected data were automatically encrypted and stored on the principal investigator’s computer to ensure privacy and confidentiality.

### Measures

#### Rate of workplace violence

Workplace violence was assessed using a dichotomous question (yes or no): “Have you encountered any workplace violence in the past 12 months?”.

#### Trivialization of workplace violence scale

Nurses’ perceptions of workplace violence were assessed using the trivialization of the workplace violence scale [36]. The scale consists of three items related to the normalization of severe violence and judgment from colleagues and employers, and each item was evaluated on a 4-point Likert scale (0 = “not at all” and 3 = “completely”). This study dichotomized this variable to measure the normalization of workplace violence (no = “not at all”; yes = “all other responses”) [36]. The Korean version was prepared using transcultural adaptation and pilot testing. Cronbach’s α coefficient for the total score was .81 in Geoffrion et al. [36], and it was .81 in our study.

#### Perception of aggression scale (POAS)

Nurses’ attitudes of aggression were measured using the Perception of Aggression Scale (POAS) [37, 38]. The POAS consists of 12 items and comprises three subscales (i.e., dysfunctional/undesirable phenomenon, functional/comprehensible phenomenon, and protective measure) [37–40]. Each item was measured on a 5-point Likert scale (1 = “strongly disagree” and 5 = “strongly agree”). A higher score indicates a more positive appraisal for aggression by a patient (range 12–60). Cronbach’s α coefficients for the dysfunctional/undesirable phenomenon, functional/comprehensible phenomenon, and protective measure were between .76 and .83 in Wong and Chien [40] and .76 and .92 in Nam et al. [37], whereas they were .84 and .90 in our study.

#### Brief coping orientation to problems experienced inventory (Brief-COPE)

Nurses’ coping styles were assessed using the Brief Coping Orientation to Problems Experienced Inventory (Brief-COPE) [41] and its Korean version after translating and conducting cross-cultural adaptation and a pilot test. The Brief-COPE consists of 28 items; each item was measured on a 4-point Likert scale (1 = “I haven’t been doing this at all” to 4 = “I’ve been doing this a lot”). By summing two relevant questions, the following 14 subscales were created: self-distraction, active coping, denial, substance use, use of emotional support, use of informational support, behavioral disengagement, venting, positive reframing, planning, humor, acceptance, religion, and self-blame; and a higher score indicates greater adoption of specific coping styles. Cronbach’s α coefficients for the 14 coping styles were between .50 and .90 in Carver et al. [41], whereas they were between .84 and .86 for South Korean nurses and between 0.87 and .89 for Chinese nurses in Hong Kong in our study.

#### Post-traumatic cognitions inventory (PTCI)

Nurses’ trauma-related thoughts and beliefs were assessed using the Post-Traumatic Cognitions Inventory (PTCI) [42] and its Korean version [43]. The PTCI consists of 33 items, and each item was measured on a 7-point Likert scale (1 = “totally disagree” to 7 = “totally agree”). A higher score indicated more frequent thoughts on negative cognition. The PTCI has three subscales, including negative cognitions about self and the world and self-blame [42]. The Korean version has five subscales, including negative self-concept about future/values, negative beliefs about others and the world, self-blame, and negative self-conception of traumatic reactions and coping styles [43]. Cronbach’s α coefficients for the total score were .95 in Cho [28] and .97 in Foa et al. [42], whereas it was .97 in our study.

#### PTSD checklist civilian version (PCL-C)

Post-traumatic stress symptoms of nurses were assessed using the Post-Traumatic Stress Disorder Checklist Civilian Version (PCL-C) [44] and its Korean version [45]. The PCL-C consists of 17 items, and each item was evaluated on a 5-point Likert scale (1 = “not at all” and 5 = “extremely”). The total score ranges from 17 to 85, with a higher score indicating more severe post-traumatic stress symptoms. The cutoff score was 44 for the general population in the PCL-C [44] and 56 for the North Korean defectors in the Korean PCL-C [45]. Cronbach’s α coefficients for the total score were .97 in Norris and Hamblen [46] and .93 in Oh et al. [45], whereas it was .97 in our study.

#### Post-traumatic growth inventory (PTGI)

Nurses’ post-traumatic growth was assessed using the Post-Traumatic Growth Inventory (PTGI) [47] and its Korean version [48]. The PTGI consists of 21 items, and each item was measured on a 6-point Likert scale (0 = “I did not experience this change as a result of my crisis” and 5 = “I experienced this change to a very great extent as a result of my crisis”). The PTGI has five subscales (i.e., relating to others, new possibilities, personal strength, spiritual change, and appreciation of life), and the Korean PTGI has four subscales (i.e., changes of self-perception, the increase of interpersonal depth, discovery of new possibilities, and the increase of spiritual interest). A higher score indicates more experience of positive change (a range of 0 to 105). Cronbach’s α coefficients for the total score were .90 in Tedeschi et al. [47] and .94 in Song et al. [48], whereas it was .96 in our study.

#### Depression anxiety stress scales short form (DASS-21)

Nurses’ negative emotional states were assessed using the Depression Anxiety Stress Scales – Short Form (DASS-21) [49] and its Korean version [50]. The DASS-21 consists of 21 items and comprises three subscales including depression, anxiety, and stress. Each item was assessed on a 4-point Likert scale (0 = “did not apply to me at all” to 3 = “applied to me very much, or most of the time”). The scores for the depression, anxiety, and stress subscales were determined by adding all items in each scale [50] and then multiplying the obtained scale scores by two [49]. A higher score indicates a greater negative emotional state of depression, anxiety, and stress. Cronbach’s α coefficients for the depression, anxiety, and stress subscales were .94, .87, and .91 in Antony et al. [51] and .87, .83, and .83 in Jun et al. [50], respectively, whereas they were .92, .91, and .91 in our study.

### Data analysis

Chi-squared or independent t-tests were used to compare demographic characteristics, perception of workplace violence, and attitudes toward workplace violence between South Korean and Chinese nurses in Hong Kong in the study sample. If the nurses answered “yes” to the question “Have you encountered any workplace violence in the past 12 months?” they were classified as “exposed.” The nurses were classified as “nonexposed” if they answered “no” to this question. After selecting nurses who have experienced workplace violence, an independent t-test was conducted to identify the mean difference between the two groups, including coping styles, post-traumatic responses (cognition, stress symptoms, and growth), and mental health indicators (depression, anxiety, and stress). The main analysis is a binary logistic regression for determining factors associated with the exposure to workplace violence in each regional group. We included independent variables that were statistically significant in the univariate analyses of at least one group. The data were analyzed using the IBM SPSS version 25 with a significance level of .05.

## Results

### Characteristics of study participants

A total of 578 South Korean nurses (*n* = 377) and Chinese nurses in Hong Kong (*n* = 201) were recruited from online communities for nurses. Of these, 471 South Korean nurses (*n* = 319) and Chinese nurses in Hong Kong (*n* = 152) consented to participate in the study and completed the survey. Study results for South Korea indicated an 84.6% response rate, a 5.0% missing rate, and a 100% completion rate, and Hong Kong indicated a 76% response rate, a 0.7% missing rate, and a 100% completion rate. Subsequently, South Korean and Chinese nurses were classified into exposed (*n* = 98, 30.7% and *n* = 48, 31.6%, respectively) and nonexposed (*n* = 221, 69.3% and *n* = 104, 68.4%, respectively) groups.

Sociodemographic and occupational characteristics of the South Korean and Chinese nurses in Hong Kong are shown in Table 1. South Korean nurses were of average age 32.64 ± 5.56 (age range 23–53), 96.2% were female, 68% held a bachelor’s degree, 86.2% (the majority) were staff nurses, and 46.7% had worked for less than 5 years. Similarly, Chinese nurses in Hong Kong were of average age 30.31 ± 6.35 (age range 22–54), 83.6% were female, 53.3% held a bachelor’s degree, 87.5% (the majority) were staff nurses, and 53.3% had worked for less than 5 years. South Korean nurses had statistically different levels of total clinical experience between the exposed and nonexposed (*p* = .044) groups, but this was not seen for Chinese nurses in Hong Kong (see Table 1).

**Table 1:** Demographic characteristics of the study participants

Variables	Categories	South Korean nurses (*N* = 319)				Chinese nurses in Hong Kong (*N* = 152)			
		Exposed group (*n* = 98)	Non-exposed group (*n* = 221)			Exposed group (*n* = 48)	Non-exposed group (*n* = 104)		
		n (%) M ± SD	n (%) M ± SD	t/χ^2^	*p*	n (%) M ± SD	n (%) M ± SD	t/χ^2^	*p*
Age (unit: year)		32.92 ± 6.02	32.51 ± 5.36	0.603	.547	30.88 ± 6.56	30.05 ± 6.26	0.746	.457
Sex	Female	94 (95.9)	213 (96.4)	0.040	.842	38 (79.2)	89 (85.6)	0.982	.322
	Male	4 (4.1)	8 (3.6)			10 (20.8)	15 (14.4)		
Marital status	Not married	57 (58.2)	132 (59.7)	0.069	.793	34 (70.8)	84 (80.8)	1.867	.172
	Married	41 (41.8)	89 (40.3)			14 (29.2)	20 (19.2)		
Religion	Yes	40 (40.8)	97 (43.9)	0.262	.609	16 (33.3)	29 (27.9)	0.468	.494
	No	58 (59.2)	124 (56.1)			32 (66.7)	75 (72.1)		
Education level	Diploma	21 (21.4)	38 (17.2)	3.189	.203	5 (10.4)	10 (9.6)	0.952	.621
	Bachelor's degree	60 (61.2)	157 (71.0)			28 (58.3)	53 (51.0)		
	Master's degree	17 (17.4)	26 (11.8)			15 (31.3)	41 (39.4)		
Hospital type	Clinic	3 (3.1)	14 (6.3)	2.284	.516	1 (2.1)	8 (7.7)	3.876	.275
	Hospital	29 (29.6)	58 (26.2)			34 (70.8)	73 (70.2)		
	General hospital	38 (38.8)	77 (34.8)			5 (10.4)	14 (13.5)		
	Tertiary care hospital	28 (28.5)	72 (32.7)			8 (16.7)	9 (8.6)		
Position	Staff nurse	87 (88.8)	188 (85.1)	1.012	.603	42 (87.5)	91 (87.5)	2.287	.319
	Charge nurse	9 (9.2)	29 (13.1)			5 (10.4)	13 (12.5)		
	Unit manager	2 (2.0)	4 (1.8)			1 (2.1)	0		
Type of work	Shift	81 (82.7)	173 (78.3)	0.800	.371	42 (87.5)	90 (86.5)	0.027	.871
	Fixed	17 (17.3)	48 (21.7)			6 (12.5)	14 (13.5)		
Total clinical experience (unit: year)	< 1	4 (4.1)	6 (2.7)	8.114	.044	3 (6.3)	4 (3.8)	0.618	.892
	1 ~ < 5	52 (53.1)	85 (38.5)			23 (47.9)	51 (49.0)		
	5 ~ < 10	20 (20.4)	75 (33.9)			12 (25.0)	24 (23.1)		
	≥ 10	22 (22.4)	55 (24.9)			10 (20.8)	25 (24.1)		

### Comparison of perception and attitudes to workplace violence

Significant differences in the perceptions of workplace violence subscales between the exposed and nonexposed subgroups for both regional groups were observed. The “exposed group” in both regions perceived that severe violence was normal, part of the job, and was not encouraged to report (*p* < .001). Hong Kong nurses only showed different attitudes toward workplace violence, such as aggression as a dysfunctional/undesirable phenomenon between the exposed and nonexposed groups (*p* = .035), whereas this was not seen for Korean nurses (see Table 2).

**Table 2:** Different perception and attitudes of workplace violence

Variables	Categories	Korean nurses (*N* = 319)				Hong Kong nurses (*N* = 152)			
		Exposed group (*n* = 98)	Non-exposed group (*n* = 221)			Exposed group (*n* = 48)	Non-exposed group (*n* = 104)		
		n (%) M ± SD	n (%) M ± SD	t/χ^2^	*p*	n (%) M ± SD	n (%) M ± SD	t/χ^2^	*p*
Perception of workplace violence	Severe violence is normal in my workplace, it is part of the job								
	No	49 (50.0)	200 (90.5)	65.00	< .001	20 (41.7)	72 (69.2)	10.44	.001
	Yes	49 (50.0)	21 (9.5)			28 (58.3)	32 (30.8)		
	Perceived taboo of complaining about workplace violence								
	No	10 (10.2)	123 (55.7)	57.70	< .001	9 (18.8)	51 (49.0)	12.61	< .001
	Yes	88 (89.8)	98 (44.3)			39 (81.3)	53 (51.0)		
Attitudes toward workplace violence	Aggression as dysfunctional /undesirable phenomenon	9.69 ± 2.90	10.26 ± 3.87	-1.30	.194	11.81 ± 3.96	13.49 ± 4.75	-2.13	.035
	Aggression as functional /comprehensible phenomenon	6.49 ± 3.72	6.95 ± 3.68	-1.04	.300	9.54 ± 3.43	9.39 ± 3.28	0.25	.800
	Aggression as protective measure	3.70 ± 2.16	3.60 ± 1.98	0.41	.679	5.94 ± 1.93	5.66 ± 1.85	0.84	.404

### Comparison of study variables between groups exposed to workplace violence

There were significant differences in post-traumatic stress, the coping styles subscales, and the negative emotional states subscales in the groups exposed to workplace violence. The South Korean exposed group had a higher score of post-traumatic stress and negative emotional states compared to the Hong Kong exposed group. Moreover, there were no differences regarding post-traumatic cognitions and post-traumatic growth in the exposed groups (see Table 3).

**Table 3:** Coping styles and post-traumatic responses of the exposed groups

Variables	Categories	South Korean nurses exposed group (*n* = 98)	Chinese nurses in Hong Kong exposed group (*n* = 48)		
		M ± SD	M ± SD	t	*p*
Coping styles	Self-distraction	6.10 ± 1.34	5.44 ± 1.29	2.858	.005
	Active coping	6.19 ± 1.49	5.52 ± 1.20	2.930	.004
	Denial	4.17 ± 1.75	3.42 ± 1.57	2.534	.012
	Substance use	4.48 ± 1.99	3.08 ± 1.89	4.047	< .001
	Use of emotional support	6.05 ± 1.54	5.85 ± 1.49	0.733	.465
	Use of instrumental support	5.97 ± 1.57	5.69 ± 1.65	1.002	.318
	Behavioral disengagement	4.12 ± 1.55	4.06 ± 1.46	0.224	.823
	Venting	5.42 ± 1.61	5.52 ± 1.66	-0.357	.721
	Positive reframing	5.71 ± 1.41	5.50 ± 1.43	0.860	.391
	Planning	5.84 ± 1.46	5.46 ± 1.29	1.531	.128
	Humor	3.57 ± 1.75	4.29 ± 1.75	-2.334	.021
	Acceptance	5.64 ± 1.21	5.90 ± 1.51	-1.092	.277
	Religion	4.33 ± 2.18	4.23 ± 1.85	0.266	.790
	Self-blame	4.67 ± 1.66	4.50 ± 1.68	0.591	.555
Post-traumatic cognitions		104.61 ± 40.06	115.02 ± 43.64	-1.432	.154
Post-traumatic stress	Re-experiencing	12.83 ± 5.35	9.42 ± 4.50	3.805	< .001
	Avoidance	16.97 ± 7.53	12.67 ± 7.59	3.235	.002
	Arousal	13.54 ± 5.59	9.46 ± 4.93	4.306	< .001
Post-traumatic growth		49.24 ± 12.80	47.25 ± 14.68	0.842	.401
Negative emotional states					
Depression		33.55 ± 12.58	22.83 ± 9.40	5.228	< .001
Anxiety		23.16 ± 10.17	22.75 ± 8.63	0.242	.809
Stress		32.96 ± 10.76	26.25 ± 9.92	3.629	< .001

### Factors to determine the groups exposed to workplace violence

The results of binary logistic regression analysis revealed that the perception, attitudes, and coping styles were factors associated with being in the exposed group. Binary logistic regression analysis provided results after adjusting the number of year about total clinical experience. The odds of exposure to workplace violence were much higher in those for whom “severe violence is part of the job” than those from whom “severe violence is not part of the job” (South Korean nurses odds ratio [OR] = 4.184, 95% confidence interval [CI] = 1.96–8.95; Chinese nurses in Hong Kong OR = 3.531, 95% CI = 1.36–9.18). The odds of exposure to workplace violence were much higher in those who “do not complain about workplace violence” than in those who “complain about workplace violence” (South Korean nurses OR = 4.580, 95% CI = 1.93–10.88; Chinese nurses in Hong Kong OR = 3.446, 95% CI = 1.13–10.56). Positive appraisal for aggression by patients was associated with the South Korean exposed group (OR = 0.905, 95% CI = 0.82–1.00). Coping styles were associated with the workplace violence experienced by Chinese nurses in Hong Kong (OR = 1.084, 95% CI = 1.03–1.14). The model accounted for 51.7% of South Korean nurses and 35.1% of Chinese nurses in Hong Kong (see Table 4).

**Table 4:** Factors associated with the workplace violence exposed groups

Variables	Categories	Korean nurse (*N* = 319)			Hong Kong nurse (*N* = 152)		
		OR	95% CI	*p*	OR	95% CI	*p*
Perception of workplace violence	Severe violence is normal in my workplace, it is part of the job						
	No (reference group)						
	Yes	4.184	1.96–8.95	< .001	3.531	1.36–9.18	.010
	Perceived taboo of complaining about workplace violence						
	No (reference group)						
	Yes	4.580	1.93–10.88	.001	3.446	1.13–10.56	.030
Attitudes toward workplace violence	Aggression as dysfunctional /undesirable phenomenon	0.905	0.82–1.00	.042	0.965	0.86–1.08	.535
	Aggression as protective measure	0.961	0.82–1.13	.632	0.937	0.74–1.18	.579
Coping styles		1.011	0.98–1.04	.503	1.084	1.03–1.14	.002
Post-traumatic cognitions		1.004	0.99–1.02	.558	1.002	0.98–1.02	.790
Post-traumatic stress	Re-experiencing	1.107	0.98–1.26	.113	0.838	0.67–1.06	.133
	Avoidance	1.020	0.92–1.13	.719	0.982	0.85–1.14	.818
	Arousal	1.059	0.93–1.21	.389	1.119	0.88–1.42	.356
Post-traumatic growth		0.987	0.96–1.01	.318	0.994	0.96–1.03	.756
Depression anxiety stress	Depression	0.975	0.90–1.06	.547	0.905	0.80–1.02	.105
	Anxiety	0.962	0.88–1.05	.404	0.981	0.89–1.08	.699
	Stress	1.064	0.98–1.16	.145	1.061	0.96–1.18	.261
Model fits		Cox & Snell *R*^2^ = .366			Cox & Snell *R*^2^ = .250		
		Nagelkerke *R*^2^ = .517			Nagelkerke *R*^2^ = .351		

## Discussion

Our study findings compared a significant association between workplace violence and post-traumatic responses through this collaborative study in South Korea and Hong Kong. The exposed group commonly perceived that severe violence was a normal part of the job and that they were not encouraged to report it. The perception, attitudes, and coping styles were differently associated factors of the exposure to workplace violence between South Korea and Hong Kong. South Korean nurses with exposure to workplace violence reported higher levels of post-traumatic stress and negative emotion compared to the Chinese nurses in Hong Kong. Thus, nursing leaders should improve the perception, attitude, and coping styles of nurses through violence-prevention education and a systematic support system. These findings expand on the definitions of workplace violence in nursing; workplace violence can be defined as a traumatic event that causes post-traumatic responses in nurses.

Previous studies have emphasized that the victim’s subjective perception of violence is more important than the violent situation itself [13, 52, 53]. Workplace violence against nurses tends to be underreported; researchers reported that it may be related to nurses’ perceptions of workplace violence [1, 13, 54, 55]. Nurses who are repeatedly exposed to workplace violence tend to give up on their efforts to change their situation [52, 54, 55]. Our study’s findings suggest that it is most important for nurses to recognize workplace violence as unacceptable and that they must be protected from workplace violence. Therefore, nursing leaders must be able to correctly recognize workplace violence against their nursing workforce [12, 13, 24, 25] and develop support policies that ensure “zero tolerance” against violence in the workplace [1, 56, 57].

In our study, attitude scores of Chinese nurses in Hong Kong were higher than those of the South Korean nurses, and attitudes toward workplace violence against Chinese nurses in Hong Kong were significantly different between the exposed and nonexposed groups. Our study findings indicate a more positive appraisal among Chinese nurses in Hong Kong of patients’ aggression than South Korean nurses. These findings are consistent with previous study results showing that patient aggression leads to physical and psychological damages on nurses and inhibits nursing professionals’ identity development [22, 24, 58–61]. Thus, researchers have tried to develop prevention and management tactics for patient violence, including strategies for changing attitudes toward violence [22, 24, 58–61]. Understanding nurses’ attitudes toward patient aggression can provide the evidence required to develop efficient guidelines regarding patient violence and to implement strategies for a safe work environment in Hong Kong [22, 24, 58–61]. Therefore, this study’s findings suggest that nursing leaders should make further efforts to change general nurses’ attitudes toward workplace violence by attending “management of violence” workshops and to improve their proactive attitudes against patient aggression [1, 3, 22, 59].

Based on this conceptual framework, we expected that coping styles were associated with nurses’ workplace violence. Coping styles were associated with workplace violence against Chinese nurses in Hong Kong but not with that against South Korean nurses. This finding may have some important implications for workplace violence interventions for South Korean and Chinese nurses in Hong Kong. First, our findings showed that the coping styles of South Korean nurses may be related to underestimating or not recognizing the problem of violence. This result supports previous studies showing that nurses with a greater perception of coping with workplace violence were found to be more successful in their struggle with violence [12, 13, 28, 37, 62, 63]. Therefore, workplace violence training programs for identifying the patterns of the workplace violence process can help to improve nurses’ coping styles [12, 13, 28, 37, 62, 63]. Second, this study found that Chinese nurses in Hong Kong are using coping styles in violent situations. Coping styles can help de-escalate the conflict caused by problems related to violence, but an ineffective coping style may not lead to improved violence problems [12, 13, 28, 62, 63]. Therefore, it is necessary to provide effective coping programs or resources against workplace violence [12, 13, 28, 62, 63].

No comparative studies on the mental health of South Korean and Chinese nurses in Hong Kong have been done, and, therefore, it is difficult to use our study as a comparison. In this study, the South Korean nurses who experienced workplace violence reported higher levels of post-traumatic stress and negative emotion compared to the Chinese nurses in Hong Kong. However, this finding is in line with that of previous studies showing that exposure to workplace violence was associated with a high level of post-traumatic stress in South Korean nurses [3, 4, 12, 13, 64]. More than 60% of South Korean nurses regularly experience verbal violence at work [3, 65], which can be more difficult to detect than physical forms of violence, as there are no visible signs of damage, and such violence is subtle [5]. However, 44.6% of Chinese nurses in Hong Kong reported they had experienced workplace violence, including verbal abuse/bullying (39.2%), more than physical assault (22.7%), and sexual harassment (1.1%) [22]. Therefore, South Korean nurses are less likely to perceive and report verbal violence, which goes on to adversely affect their mental health [12, 65]. However, the policy of the Ministry of Health and Welfare of South Korea places a relatively low focus on nurses’ mental health. In contrast, Hong Kong has made diverse efforts to provide training to nurses for improving their mental health. Chinese nurses in Hong Kong are provided with stress management and relaxation skills and psychological health training to protect their mental health from the impact of workplace violence [66]. Therefore, it is important to develop various mental health training programs to address the impact of verbal violence on South Korean nurses’ mental health [4, 13, 24, 64, 65, 67].

In this study, we found that the South Korean nurses had statistically significant total clinical experience differences between the exposed and nonexposed groups. This finding is consistent with previous research that found newly graduated nurses are more vulnerable to workplace violence than their older counterparts [12, 13, 22, 68].

Newly graduated nurses are strictly trained by their seniors to play new roles and adapt to nursing practice, and due to their inexperience, they are not adequately prepared to identify or prevent potential workplace violence [12, 17, 69, 70]. In situations where newly graduated nurses face workplace violence, they find it more difficult to cope than senior nurses [12, 69, 70]. Further, many studies have shown that experiences of violence are related to negative patient outcomes [17, 69, 71]. When the victim of violence is a newly graduated nurse, negative patient outcomes are likely to be more serious. Therefore, this study’s findings suggest that workplace violence against newly graduated nurses should be managed with great care, and sufficient training should be provided to build resilience [66, 68, 72, 73].

### Implications

In this study, we recommend that workplace violence against nurses can be prevented by improving their working environment [1, 3, 66]. In our sample, nurses reported a lack of organizational prevention actions for reducing violence [52]. This indicates that organizations have a lack of perception of the seriousness of workplace violence and do not have an effective stress management system [1, 53]. Nurses’ low perception of workplace violence can reduce their work performance, organizational commitment, and confidence within nursing practice, which, in turn, can negatively affect patients [1, 52]. Therefore, organizations should establish a systematic reporting system and improve not only the perceptions of nurses but also those of patients, patient families, and other health care workers through workplace violence–prevention campaigns [1, 74]. Finally, the findings suggest that cultural differences are related to workplace violence between South Korean and Chinese nurses in Hong Kong. A culture of health care provides the foundation for building a safe supportive work environment in the nursing field [14–16]. Therefore, nursing leaders should consider cultural or organizational influences to understand the phenomena of workplace violence and develop effective interventions to cope with workplace violence in different settings [14–16]. To mitigate workplace violence against nurses, further research is needed that compares various cultures.

### Limitations

This study has several limitations. First, data from South Korea were collected at an early stage of the COVID-19 pandemic, whereas those from Hong Kong were collected when COVID-19 had already spread worldwide. This may have resulted in different impacts on nurses who experienced workplace violence in these two regions. A previous qualitative study referred to the impact of the COVID-19 pandemic on nurses in Hong Kong, including frequent resource shortages, changing nursing protocols, and physical and mental health threats. Our study, which is the first study in Hong Kong, reported the impact of the COVID-19 pandemic on nurses in Hong Kong. Second, the Hong Kong sample size was smaller than the South Korean one; thus, caution must be maintained while generalizing the findings of this study. Third, some of the English questionnaires were translated into South Korean, which was then validated using a cross-sectional online survey. These questionnaires could not be verified with greater sample sizes and in diverse working environments because of time constraints. Finally, it would be beneficial to expand this study, in combination with a qualitative research, in order to integrate a cultural context into the understanding of workplace violence in specific environments and different nursing systems [75].

## Conclusions

We conducted a comparative study between South Korean and Chinese nurses in Hong Kong who have experienced workplace violence in terms of the processes and outcome factors related to this type of violence. Our findings confirmed that nurses of these two regions have somewhat different perceptions, attitudes, and coping styles related to post-traumatic responses prompted by workplace violence. The findings highlighted the need to encourage zero tolerance of workplace violence, develop a more active attitude toward workplace violence, and increase the use of coping styles at the individual level. Furthermore, organizational support and systemic workplace violence prevention are needed for cultivating a safe working environment and better nursing professionalism.

## Data Availability

Data and materials are available at the request of the readers. Requests for data should be addressed to the corresponding author.

## Abbreviations

COVID-19: Coronavirus Disease of 2019
POAS: Perception of Aggression Scale
Brief-COPE: Brief Coping Orientation to Problems Experienced Inventory
PTCI: Post-Traumatic Cognitions Inventory
PCL-C: PTSD Checklist Civilian Version
PTGI: Post-Traumatic Growth Inventory
DASS-21: Depression Anxiety Stress Scales Short Form
OR: Odds ratio
CI: Confidence interval
DASS-21: Depression Anxiety Stress Scales-Short Form
